# Localised increase in regional cerebral perfusion in patients with visual snow syndrome: a pseudo-continuous arterial spin labelling study

**DOI:** 10.1136/jnnp-2020-325881

**Published:** 2021-07-14

**Authors:** Francesca Puledda, Christoph J Schankin, Owen O'Daly, Dominic Ffytche, Ozan Eren, Nazia Karsan, Steve C R Williams, Fernando Zelaya, Peter J Goadsby

**Affiliations:** 1 Headache Group, Wolfson CARD, Institute of Psychiatry, Psychology & Neuroscience, King’s College London, King's College London, London, UK; 2 NIHR-Wellcome Trust King’s Clinical Research Facility, SLaM NIHR Biomedical Research Centre, King's College Hospital, London, UK; 3 Department of Neurology, Inselspital University Hospital Bern, Bern, Switzerland; 4 Centre for Neuroimaging Sciences, Department of Neuroimaging, Institute of Psychiatry, Psychology and Neuroscience, King's College London, London, UK; 5 Old Age Psychiatry, Institute of Psychiatry, Psychology and Neuroscience, King's College London, London, UK; 6 Department of Neurology, University Hospital Munich Campus Grosshadern, Munchen, Germany

## Abstract

**Objectives:**

We aimed to investigate changes in regional cerebral blood flow (rCBF) using arterial spin labelling (ASL) in patients with visual snow syndrome (VSS), in order to understand more about the underlying neurobiology of the condition, which remains mostly unknown.

**Methods:**

We performed an MRI study in which whole-brain maps of rCBF were obtained using pseudo-continuous ASL. Twenty-four patients with VSS and an equal number of gender and age-matched healthy volunteers took part in the study. All subjects were examined with both a visual paradigm consisting of a visual-snow like stimulus, simulating key features of the snow, and a blank screen at rest, randomly presented.

**Results:**

Patients with VSS had higher rCBF than controls over an extensive brain network, including the bilateral cuneus, precuneus, supplementary motor cortex, premotor cortex and posterior cingulate cortex, as well as the left primary auditory cortex, fusiform gyrus and cerebellum. These areas were largely analogous comparing patients either at rest, or when looking at a ‘snow-like’ visual stimulus. This widespread, similar pattern of perfusion differences in either condition suggests a neurophysiological signature of visual snow. Furthermore, right insula rCBF was increased in VSS subjects compared with controls during visual stimulation, reflecting a greater task-related change and suggesting a difference in interoceptive processing with constant perception of altered visual input.

**Conclusion:**

The data suggest VSS patients have marked differences in brain processing of visual stimuli, validating its neurobiological basis.

## Introduction

Visual snow is a neurological condition defined by the presence of a continuous and unremitting visual disturbance in the form of uncountable tiny dots covering the whole visual field.^1^ Patients affected by visual snow syndrome (VSS) experience a complex array of neurological and visual symptoms in addition to the static itself, such as palinopsia, entoptic phenomena, nyctalopia and photophobia.^2^ Visual snow represents a spectrum type disorder that at its worse manifests with most of these additional symptoms, as well as with distressing comorbidities such as migraine and tinnitus^3^; in such cases, the condition is perceived as very disabling.^4^

Although the pathophysiology of VSS remains largely unknown^5^ a growing body of literature has started offering some insight on the possible biological mechanisms underlying the condition. Behavioural^6^ and neurophysiological studies^7 8^ have shown patterns of changes pointing to increased cortical excitability and visual cortex dysfunction. Through neuroimaging, it has been possible to determine that VSS is characterised by altered metabolism of the extrastriate visual cortex^9 10^ as well as structural changes involving the visual system, and further expanding beyond it.^9 11 12^

Arterial spin labelling (ASL) is a quantitative, non-invasive functional MRI technique that has evolved considerably in the last decade^13^; this method exploits the phenomenon of neuro-vascular coupling to use resting perfusion as an indirect but sensitive marker of neuronal activity.

In this study we investigated intrinsic differences in brain activity in patients with VSS compared with controls, determining differences in regional cerebral blood flow (rCBF) using ASL. Given that VSS is characterised by an abnormal perception, in order to differentiate changes due to altered visual processing from those of the ongoing visual snow effect itself, we studied subjects both at rest and during a visual task that simulated the visual snow experience. We hypothesised those areas in the visual network would be characterised by changes in cerebral blood flow in VSS subjects, indirectly reflecting changes in neuronal activity. A further hypothesis was that these areas would not show particular changes in different states of brain activity, since visual snow is a continuous phenomenon that does not appear to be influenced by external conditions.

## Methods

### Subject population and recruitment

Twenty-four patients with a diagnosis of VSS according to the current criteria^1^ and an equal number of age and gender matched healthy volunteers were selected for the study. This number was based on the calculation that to detect a significant two-tailed difference with 94% power (with a minimum CBF change of 5 mL per 100 g of tissue per min) between two independent groups with a SD of 8%, we would require at least eighteen subjects per group. We recruited VSS patients by email, re-approaching subjects who had previously contacted our study team asking to participate in research. The healthy controls (Ctrls) were recruited through internal advertisement at King’s College London.

Recruitment was limited to individuals of 20–60 years of age with no contraindications to MRI, no serious medical conditions, consumption of no more than six cups of coffee per day and who were naïve to any type of recreational drugs, including cannabis. Any participant taking recurrent medications with an action on the central nervous system was excluded from the study. Patients with a history of psychosis or psychological diseases either requiring ongoing psychoactive drugs, or that was thought to affect the patient’s neural pathways, were excluded from the study. Controls were selected based on matching age (±5 years) and gender of our patient population. All controls were thoroughly screened to exclude any visual snow symptoms, as well a migraine history and migraine markers, by a trained neurologist and headache specialist.

### Study protocol

The study involved a telephone interview, in which eligibility of the participant was assessed, followed by two visits to our research facility. During the first visit patients had a general and neurological examination, blood pressure and heart rate monitoring. Each patient underwent a clinical interview, focusing on VSS symptoms, medical history and migraine history. VSS diagnosis was based on the presence of visual static lasting for at least 3 months, as well as at least two additional visual symptoms among: palinopsia, entoptic phenomena, photophobia and nyctalopia.^1^

The PHQ-8 and GAD-7 questionnaires were used to assess respectively potential depression and anxiety, with clinical relevance defined by a score of above 9.^14 15^

Eligible patients were invited for a second visit in which the scanning took place; controls only came for the scanning visit. All participants were scanned at the same time of day (between 09:00 and 12:00), as it is known that circadian rhythms can influence rCBF.^16^ Subjects were instructed to consume a light breakfast and to avoid caffeine immediately prior to the visit. Participants were asked to refrain from the use of any type of medication for 24 hours prior to scanning. Female patients were asked to keep a menstruation diary for the time of the study, in order to avoid scanning on days of active menstruation. To ensure VSS patients were not scanned during an acute migraine, they were instructed to inform the investigators if a migraine attack was experienced in 48 hours prior and following the imaging visit. This was further verified during the visit itself by the investigator and by email follow-up.

### Imaging procedure

All scans were conducted in a single session on a 3T General Electric MR750 MRI scanner at the NIHR-Wellcome Trust King’s Clinical Research Facility, King’s College Hospital, London using a 12-channel head coil. The scanning protocol was the same for both groups.

High resolution three-dimensional T1-weighted images were acquired to facilitate the spatial normalisation of the CBF maps. The parameters of this scan were: repetition time (TR)=7.312 ms; echo time (TE)=3.016 ms; inversion time (TI)=400 ms; flip angle (FA)=11°; field of view (FOV)=270×270 mm; matrix=256×256; slice thickness=1.2 mm; 196 slice partitions, ASSET factor=1.75; in-plane resolution=1 mm.^17^

Whole-brain CBF maps were generated by means of a three-dimensional pseudo-continuous ASL (3D-pCASL) sequence. Labelling of arterial blood was achieved with an 1825 ms train of Hanning shaped radiofrequency pulses of 500 µs duration in the presence of a net magnetic field gradient along the flow direction (the z-axis of the magnet). After a post-labelling delay of 2025 ms, a whole-brain volume was read using a three-dimensional inter-leaved ‘stack-of-spirals’ Fast Spin Echo readout, consisting of eight interleaved spiral arms in the in-plane direction, with 512 points per spiral interleave. The images had 60 axial slice locations (3 mm thickness) and an in-plane FOV of 240×240 mm after transformation to a rectangular matrix (TE/TR=11.088/5180 ms, FA=111°). A proton density image volume with the same parameters was acquired within the same sequence in order to use as a reference to compute the CBF maps in conventional physiological units (mL blood per 100 g tissue per min). The sequence used four background suppression pulses to minimise static tissue signal at the time of image acquisition. Four control-label pairs were acquired. CBF maps were computed from the mean perfusion weighted difference image derived from the four control-label pairs, by dividing the difference image against a proton density image acquired at the end of the sequence, using identical readout parameters. This computation was done according to the formula suggested in the recent ASL consensus,^18^ and is described with the full preprocessing procedure of CBF maps in online supplemental material. The entire acquisition time of the 3D-pCASL sequence was 6 min and 20 s.

10.1136/jnnp-2020-325881.supp1 Supplementary data

All participants in the study were subject to two separate pCASL acquisitions, one at baseline and one during a visual task. During the baseline sequence, participants were lying still with their eyes open while looking at a blank screen, which they viewed through a mirror system. For the visual task sequence, participants had to view a visual task that mimicked the static of visual snow, shown continuously through the same screen. The development of the visual task has been described in detail in a previous publication by our group.^10^ Overall, this ‘snow-like’ visual simulation was evaluated as very similar to the subjects’ own snow (see online supplemental material).

### Analysis of pCASL data

Processed whole-brain CBF images were analysed using a voxel-wise general linear model in SPM 12 (www.fil.ion.ucl.ac.uk/spm/). A voxel-wise flexible-factorial design using two-way ANOVA allowed analysis of changes in CBF related to group and stimulus effect. The resulting Z statistic images were reviewed with an initial cluster-forming voxel threshold of *p*<0.001 and was family-wise error (FWE) corrected, on the basis of cluster extent, to *p*<0.05, using the Gaussian random field theory. Mean global CBF (see online supplemental material) was included as a covariate in the design matrix using ANCOVA, to account for inter-individual differences in global perfusion. All brain locations are reported as x, y and z coordinates in Montreal Neurological Institute (MNI) space; cluster size is reported as k. A neuroanatomy atlas,^19^ as well as the Harvard-Oxford cortical and subcortical structural atlases from the FSL software (FSL V.5; https://fsl.fmrib.ox.ac.uk/fsl/fslwiki), were used to identify the correct anatomical locations of clusters of statistically significant changes within MNI space.

### Comparative analysis of VSS and migraine

Migraine is an important comorbidity of VSS, being reported in over 70% of patients.^3^ This high association makes it difficult to select subjects with isolated VSS and to rule out spurious effects of migraine on investigation results. For this purpose, and to understand more about VSS and migraine comorbidity, we conducted two subanalyses in our cohort, as follows. First, we ran a validation analysis with a cohort of 24 episodic migraineurs, with no history of visual snow, enrolled in a previous pCASL imaging study performed within our group.^20^ pCASL images for this study followed the same acquisition and processing parameters as the ones described here. A one-way ANOVA design was used, comparing the three groups—VSS patients, migraine patients, healthy controls—at baseline, when not subject to visual stimulus.

We further performed a *post-hoc* group comparison for the baseline condition on subjects with VSS and no migraine history (n=9), compared with all Ctrls (n=24). Given the low power of this analysis, the initial cluster-forming threshold was lowered to *p*<0.01 and FWE corrected to *p*<0.05.

## Results

### Participant demographics

Mean age of the VSS patient group was 28±6; female:male ratio was 12:12. Ten patients had VSS for as long as they could remember, while eight reported a sudden onset of symptoms. Of these, two recalled onset with a migraine attack, one with a mild head trauma and the remaining could not associate any particular event to the onset. Fifteen patients (63%) had episodic migraine, with seven also presenting a typical aura diagnosis.^21^ Nineteen (80%) had concomitant tinnitus. The questionnaires detected four patients as having anxiety and depression, four depression only and two anxiety only. All VSS subjects reported no change in the intensity or features of their symptoms between visit 1 and 2.

Full clinical features of the patient population have been detailed elsewhere.^11^

### rCBF changes in VSS

#### Main group effect

To test our main hypothesis that patients with VSS would show areas of altered regional blood flow compared with controls, we performed a whole-brain comparison of CBF maps for the main effect of group (i.e. VSS vs. Ctrls).

This analysis showed an extensive network of unilateral and bilateral brain regions with increased rCBF in patients with VSS compared with Ctrls. Specifically, bilateral clusters of significant rCBF change were found in the cuneus, precuneus, inferior parietal lobule (IPL), superior parietal lobule (SPL), supplementary motor area, frontal eye fields (FEF), premotor cortex, posterior cingulate cortex (PCC), middle frontal gyrus, angular gyrus (AG), post central gyrus, middle and superior occipital lobule. In the left hemisphere only, areas of increased rCBF were found in the primary auditory cortex, fusiform gyrus, area VI of the cerebellum and supramarginal gyrus. Anatomic locations and descriptions of all the significant clusters are highlighted in table 1. There were no areas of reduced rCBF in VSS compared with Ctrls.

**Table 1:** Brain areas of significant differential regional cerebral blood flow increase in patients with visual snow syndrome respect to healthy controls (main effect of group), shown in coordinate Montreal Neurological Institute space with relative *T* and k values

Brain region Cluster description	Peak description	T	k	Peak coordinates		
				x	y	z
Cuneus and precuneus	Right cuneus, precuneus, IPL, SPL, AG, superior occipital lobule, post central gyrus, BA7	5.37	742	52	−54	54
Cuneus and precuneus	Left cuneus, precuneus, IPL, SPL, middle occipital lobule, AG, post central gyrus, BA7	4.7	1380	−26	−82	32
Superior temporal gyrus	Left superior temporal gyrus, primary auditory cortex, IPL, transverse temporal gyrus	4.62	219	−54	−30	14
Precentral gyrus	Left premotor cortex, FEF, middle frontal gyrus, inferior frontal gyrus, BA6	4.61	460	−46	0	46
Precentral gyrus	Right premotor cortex, FEF, middle frontal gyrus, inferior frontal gyrus, BA6	4.35	422	44	0	48
Cerebellum and fusiform gyrus	Left cerebellum, fusiform gyrus, inferior temporal gyrus	4.22	310	−38	−56	−22
Inferior parietal lobule	Left IPL, superior temporal gyrus, supramarginal gyrus, BA40	4.13	209	−64	−40	42
Posterior cingulate gyrus	Bilateral PCC, medial precuneus	4.1	696	−4	−40	46
Supplementary motor area	Bilateral SMA, superior frontal gyrus, anterior cingulate gyrus	3.82	402	2	10	52

An initial voxel threshold of *p*<0.001 and cluster correction to *p*<0.05 was applied.

AG, angular gyrus; BA, Brodmann area; FEF, frontal eye fields; IPL, inferior parietal lobule; PCC, posterior cingulate cortex; SMA, supplementary motor area; SPL, superior parietal lobule.

We then proceeded to test the group effect (VSS vs Ctrls) separately in the two conditions (ie, at baseline only and during visual stimulation only).

#### Group effects at baseline

**Figure 1:**
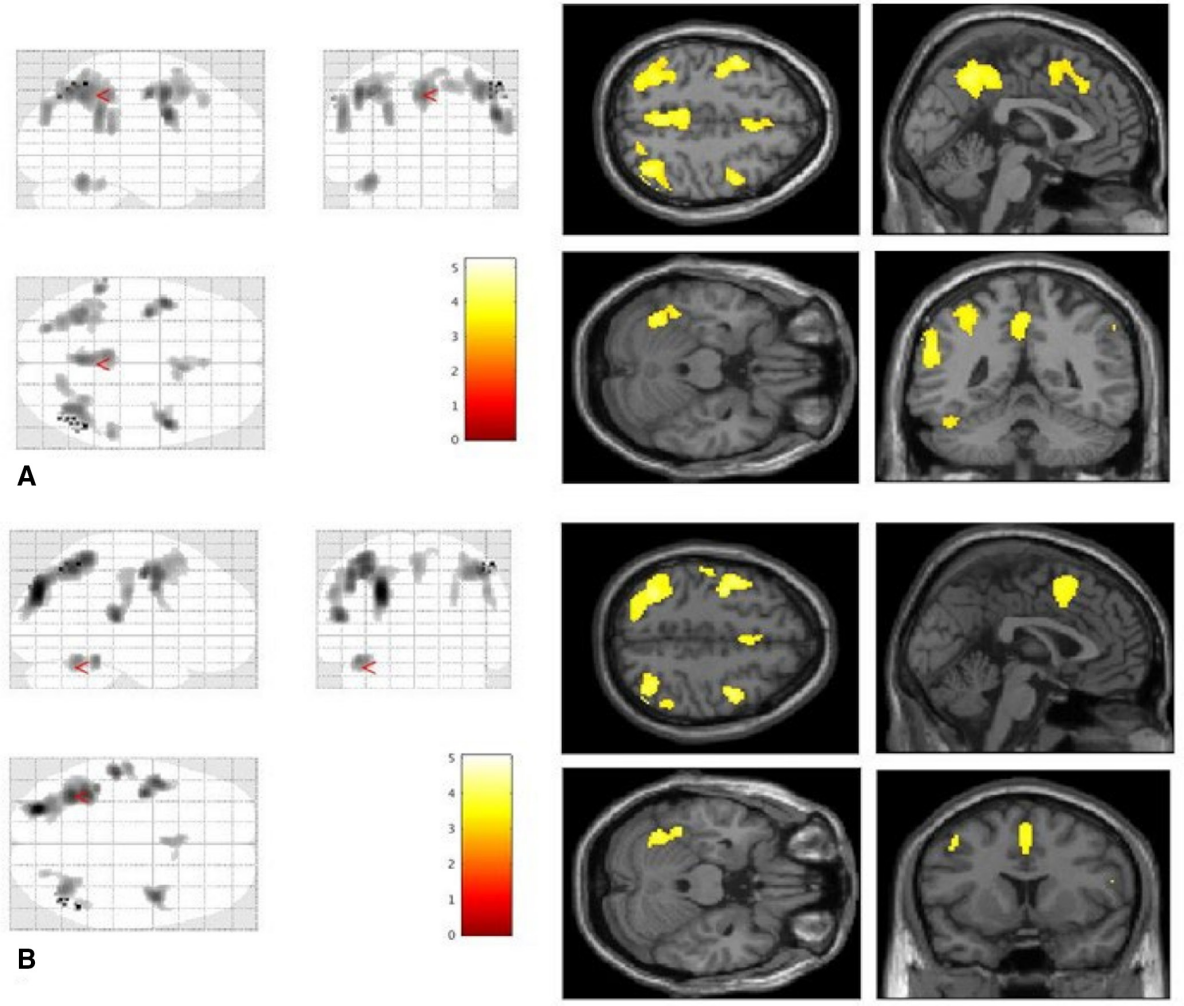
Areas of increased regional cerebral blood flow in patients with visual snow syndrome (*n*=24) compared to healthy controls (*n*=24) when looking at a blank screen at baseline (A) and when observing a ‘snow-like’ visual stimulus (B). All areas are significant at the cluster level whole-brain analyses and corrected for cluster extent. Bars represent *T* values.

When comparing whole-brain CBF maps of VSS patients to Ctrls at rest, an equal number of largely overlapping areas of increased rCBF were found (figure 1A) as the main group analysis described earlier.

#### Group effects with stimulus

When comparing VSS to Ctrls in the visually stimulated state, the areas of significant rCBF difference were found again to overlap with the main group effect analysis (figure 1B). Some minor differences related to the absence of two clusters, involving the bilateral PCC and left IPL, which failed to reach the significance threshold.

**Figure 2:**
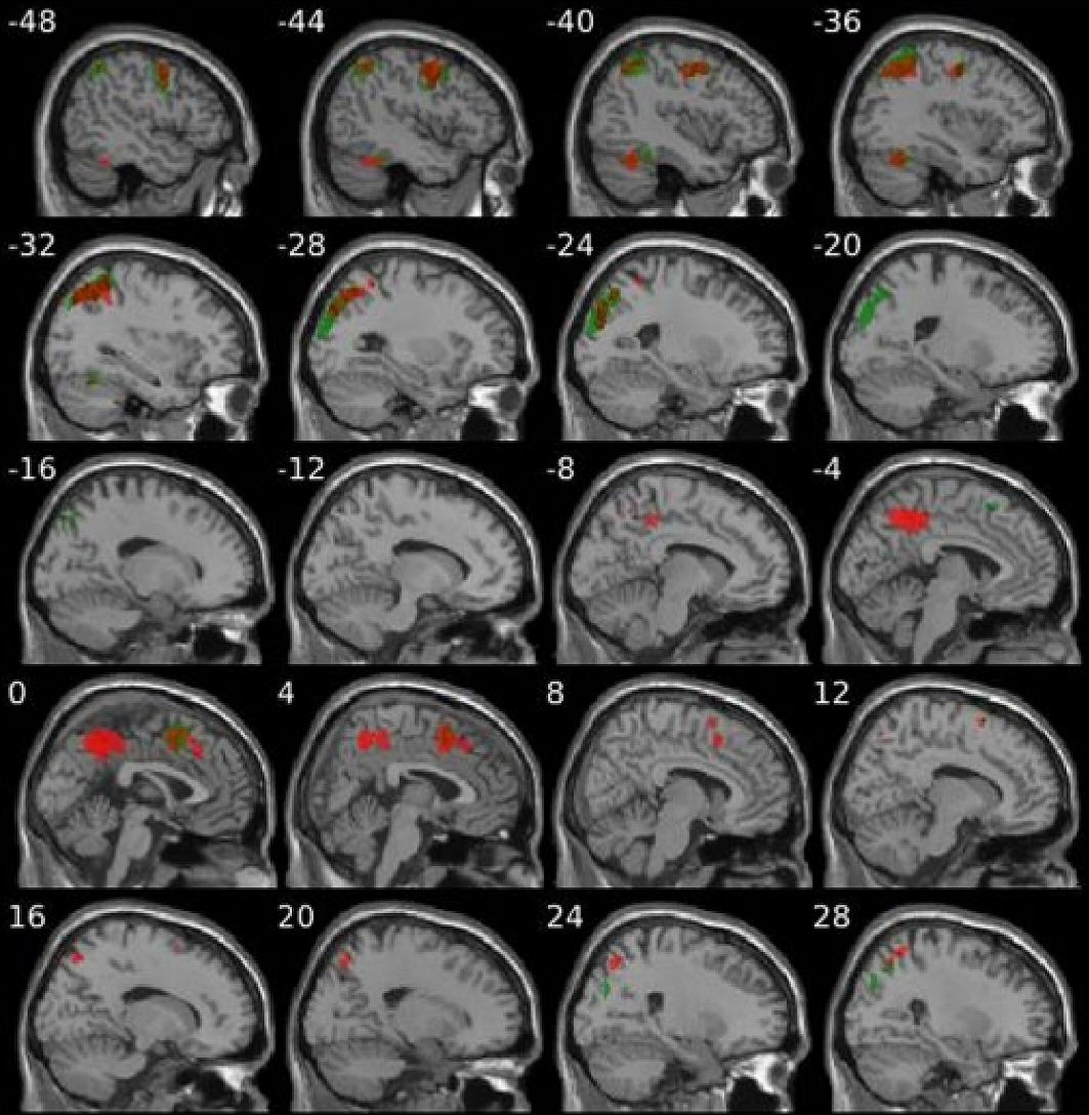
Comparative illustration of areas of increased regional cerebral blood flow in visual snow syndrome versus healthy controls when looking at a blank screen (red colour areas—as seen in figure 1A) and when observing a ‘snow-like’ visual stimulus (green colour areas—as seen in figure 1B).

The clear similarity between group differences in the two states (at rest and during the task condition) is illustrated in figure 2.

### VSS patients and stimulation: interaction effects

**Figure 3:**
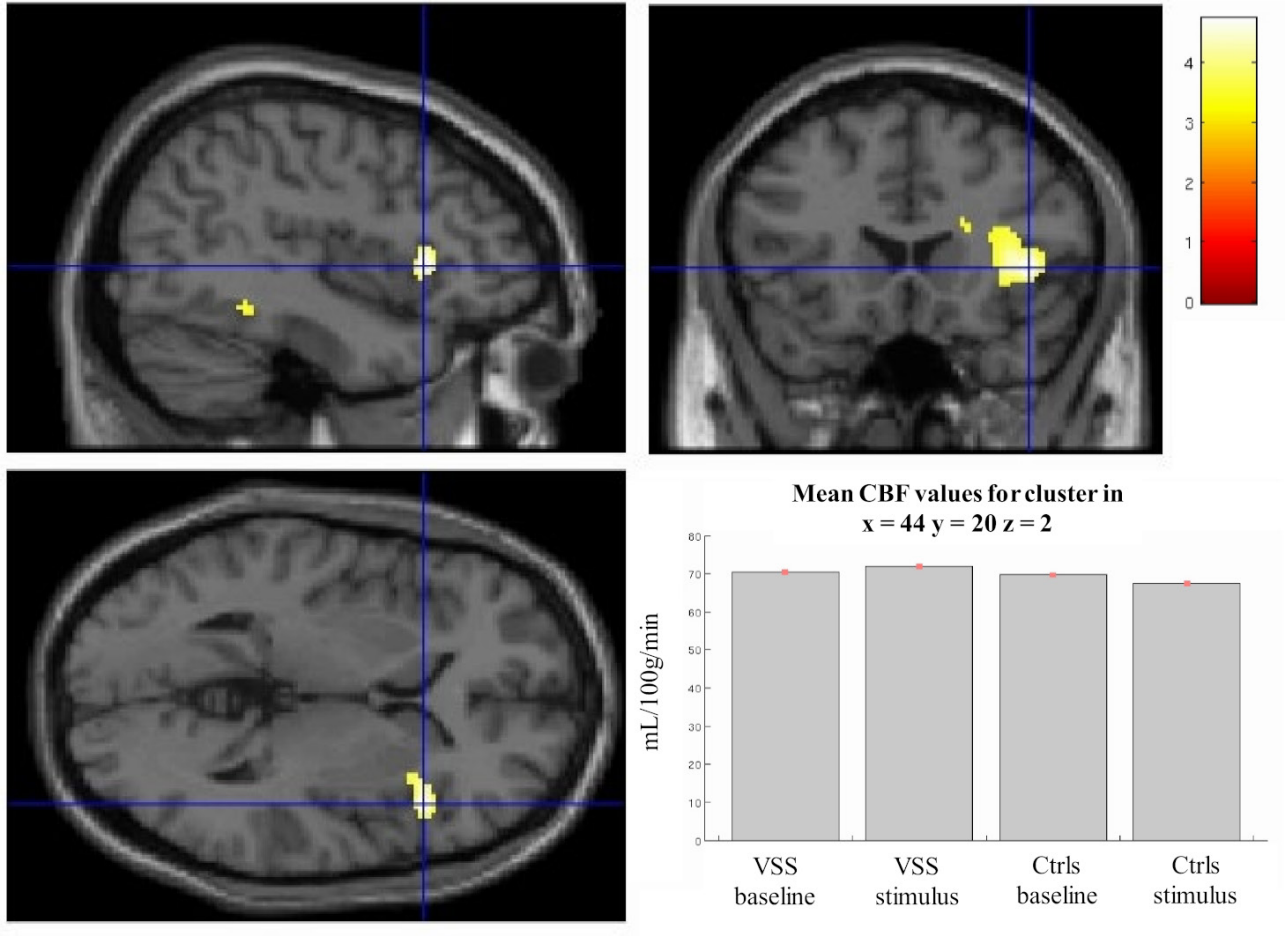
Right insula activation in patients with VSS when testing for group and stimulation interaction. This area was significant for *p*=0.01 after SVC by applying a mask over the right insula. Plots for mean CBF values for the cluster in each condition are shown in bottom right. k=98; Montreal Neurological Institute coordinates: x=44, y=20, z=2. Bar represents *T* values. CBF, cerebral blood flow; Ctrls, healthy controls; VSS, visual snow syndrome.

When considering the effect of the interaction between groups (ie, VSS vs Ctrls) and stimulus conditions (ie, baseline vs visual task), we found a significant cluster of increased task-related activation in VSS compared with Ctrls, located in the right anterior insula (x=44, y=20, z=2; figure 3). The analysis shows that, following the stimulation, there is a task-related rCBF increase in this specific brain area in the VSS group, as opposed to the deactivation seen in Ctrls.

### Comparative analysis between VSS and migraine

**Figure 4:**
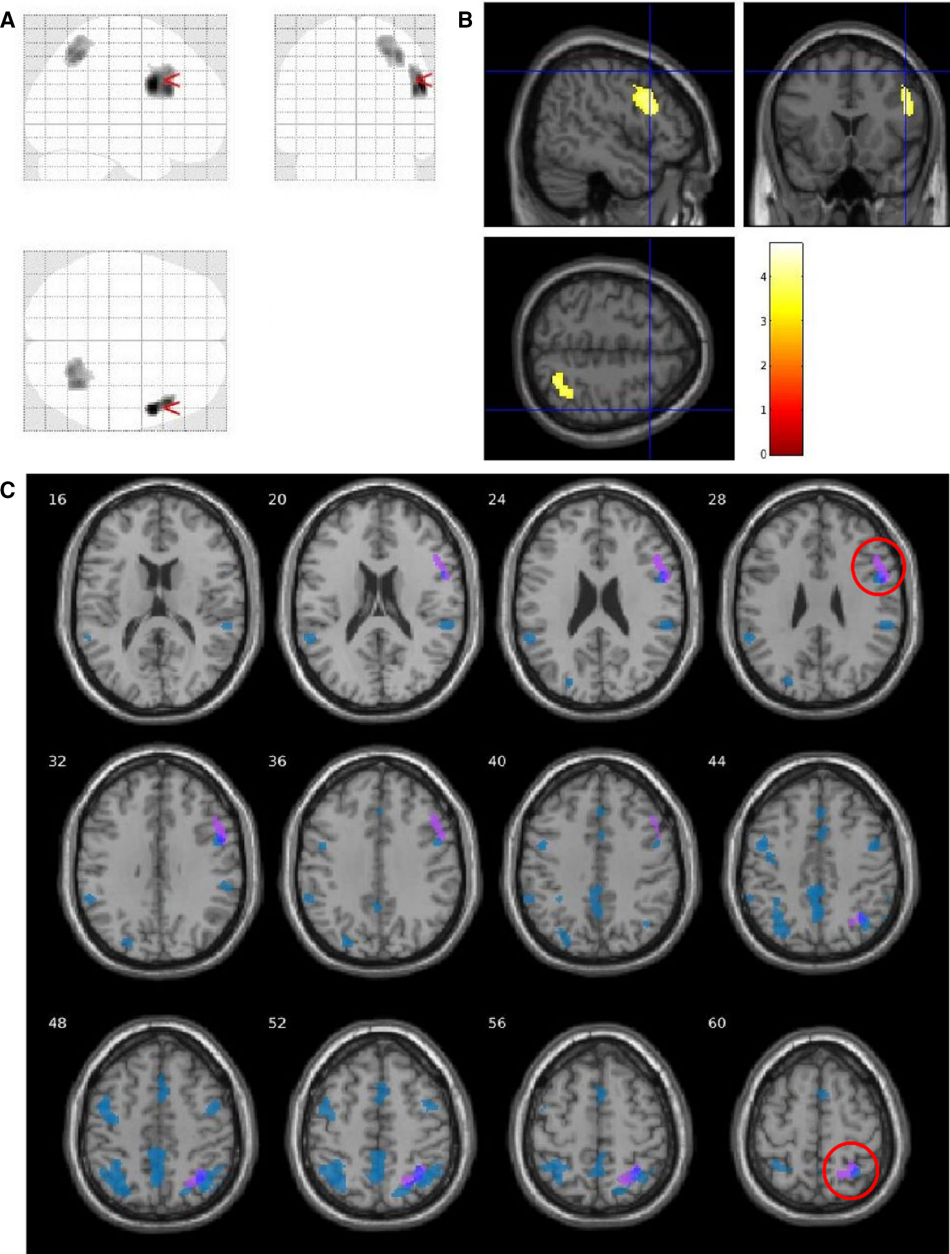
Analysis comparing visual snow syndrome (VSS) patients versus migraine patients versus healthy controls (Ctrls), showing two clusters of increased regional cerebral blood flow in VSS patients imposed over a glass brain (A) and standard T1 image (B). Clusters are located in the right precentral gyrus (x=54, y=6, z=28; *T*=4.40; k =420; *p*=0.001) and right precuneus (x=34, y=−56, z=50; *T*=4.04; k=404; *p*=0.001). A comparative image (C) with the whole-brain analysis between VSS patients and Ctrls shows that the two clusters overlap the significant areas previously found. SVC, small volume correction.

By comparing VSS patients, Ctrls (the same subjects as the main analysis) and migraine patients from a previous study, we found that two of the nine clusters of increased rCBF in VSS patients seen in the whole-brain analysis (table 1) survived; these corresponded to the right precentral gyrus (x=54, y=6, z=28; *T*=4.40; k=420) and right precuneus (x=34, y=−56, z=50; *T*=4.04; k=404; figure 4). Further, when comparing VSS patients to migraineurs directly, we found one region of increased rCBF (at a cluster-forming threshold of *p*<0.005) corresponding to the aforementioned right precuneus cluster.

When running a *post-hoc* analysis on the nine VSS subjects with no migraine history, we found a total of four clusters of significantly increased rCBF in patients with VSS compared with Ctrls. These were located in the left superior temporal gyrus (x=−52, y=−28, z=12; *T*=4.19; k=624), right SPL, cuneus and precuneus (x=28, y=−44, z=44; *T*=3.79; k=767), left PCC (x=−8, y=−42, z=40; T=3.71; k=631) and left SPL, cuneus and precuneus (x=−32, y=−58, z=48; *T*=3.36; k=930). These areas were largely overlapping the posterior clusters emerging from the original whole-brain analysis (table 1) conducted on all VSS subjects.

### Additional analyses

Analysis of global cerebral blood flow, F statistics and motion parameters in the two groups, as well as changes in regional CBF following the visual task with respect to the baseline condition (ie, stimulus effects) can be found in online supplemental material. No differences to the main effect of group analysis were found when comparing separately subjects who first received the task to subjects who first were at rest, showing no carry-over effects of the visual paradigm on brain perfusion. A *post-hoc* analysis with tinnitus presence as a covariate showed overlapping clusters with respect to the main analysis, but required lowering the significance threshold to p<0.01.

## Discussion

### Perfusion changes in VSS

Our data show that patients with VSS exhibit a specific pattern of increased regional perfusion in several brain areas, which are mostly involved in complex sensory processing, and that are not seen, by comparison, in healthy subjects. The data indicate an underlying neurobiological disturbance in VSS. Given these alterations can be seen both in the resting brain and during a specific visual task, this suggests an intrinsic pattern of altered neuronal function in VSS, which could represent a specific pathophysiological fingerprint of the condition. A confirmation of this comes from the knowledge that several of these regions have been involved in VSS pathophysiology with other neuroimaging modalities as well.

#### Parietal cortex and its functional networks

The clusters showing highest levels of rCBF increase in our analysis occupied large areas of the posterior and lateral parietal cortex, in particular the SPL, IPL, AG, precuneus and cuneus of both hemispheres, as well as the occipital cortex.

The parietal cortex has a fundamental role in the integration of different sensory stimuli.^22^ In particular with regards to visual stimulus processing, the dorsal visual stream represents a brain pathway in which visual information is delivered from the primary visual cortex to the posterior parietal lobe and onwards to other integrative areas of the brain. It is involved in the visual location of objects and is essential in determining action-oriented behaviours dependent on the perception of space.^23 24^ Elements of the dorsal visual stream showed increased rCBF in our analysis, as well as Brodmann area 7, a point of convergence between vision and proprioception, which allows to determine where objects are in relation to the body.^25 26^

It is also relevant to note that the precuneus and PCC constitute the posterior elements of the default mode network (DMN), an organised mode of brain function active when the brain is at rest and suspended during specific goal-directed behaviours.^27 28^ These areas are strongly linked to the recollection of prior experiences, involving both the external and internal world.^29^ The fact that these regions showed increased blood flow bilaterally, could potentially signify an increased function within the DMN in VSS patients; ultimately, this could be leading to a misattribution of brain energy, favouring internal experiences over external attention. This finding has some correspondence with data from a functional connectivity analysis performed in this same group of patients.^12^

Further, the sensorimotor region of the anterior precuneus has been shown to connect directly to the supplementary motor area,^30^ also exhibiting increased perfusion. These regions, as well as the angular gyrus, are typically activated in cognitively demanding tasks,^31^ in the control of internally generated or visually guided movement^32 33^ and in visuoproprioceptive integration.^34^ The more medial aspects of the precuneus are also an element of the fronto-parietal network,^35^ which specialises in external attention^36^ and visuospatial perception.^37^ The collective involvement of these brain areas could thus potentially lead, in visual snow, to an abnormal focusing on normal sensory phenomena.

#### Visual motion function

The large parietal region of increased perfusion encompasses visual area V5, a brain region that specialises in processing and computing visual motion, by integrating and decoding inputs it receives from the primary visual cortex.^38 39^ An increased function of this region justifies its larger volume detected through structural imaging in VSS^11^ and is certainly consistent with the misperception of constantly moving objects typical of the condition. However, in order to assess fully the role of V5 in VSS, specific tasks related to visual motion perception would be needed in the future.

The frontal eye fields also have a significant role in visual motion and visuo-spatial attention.^40^ Interestingly, the FEF have also shown abnormal connectivity to the angular gyrus in a previous functional brain study on visual snow patients.^41^ Given that both regions showed increased perfusion in our study, these findings seem to confirm changes in attentional control, as well as integration of visual movement and proprioception, in VSS.

#### Insular involvement

Our data also showed an increased activation in response to the ‘snow-like’ stimulus within the right anterior insula, in patients with VSS compared with controls (figure 3). This heightened activation was opposite to the stimulus-induced deactivation that was seen in healthy subjects.

The insular lobe of Reil has a pivotal salience function, relaying different sensory inputs to other areas of the limbic system and the brain.^42^ The right anterior insula, in particular, represents a hub capable of ‘switching’ brain engagement from the internally oriented activity of the DMN to the externally oriented regions of the executive network, which mediate attention, memory and higher order cognitive processes; this ultimately allows to determine appropriate behavioural responses to salient stimuli.^43 44^

Insular involvement in VSS pathophysiology had already been recorded by our group using functional MRI,^10^ being linked to the altered processing of an analogous visual stimulus to the one used here. Interestingly, we had previously found the insula to deactivate—in contrast to controls who showed a null activation—in response to this stimulus, rather than an increase in its activity. It must be noted, however, that even if the stimuli used in the two techniques were identical in their visual parameters, their time presentations were entirely different. Here, the MRI acquisition lasted several minutes, allowing to detect a more prolonged and sustained change in brain activity. It is thus possible that the insula exhibits altered function in VSS, possibly causing increased sensitivity of the visual and sensory pathways and altered processing of visual stimuli.

#### Cerebellum: a key player in VSS?

The posterior and lateral cerebellum, particularly of the left hemisphere, play a relevant role in complex cognitive operations linked to spatial processing, language and memory.^45 46^ Furthermore, activity in cerebellar lobule VI has shown high correlation with activation of the salience network.^47^ This, taken together with the finding of increased volume of overlapping cerebellar areas in a structural study of VSS,^11^ suggests that the cerebellum might represent a key structure in the biology of the condition, possibly involved in dysfunctional feed-forward mechanisms of sensory processing.

#### Visual and auditory cortices

Even if we did not detect specific perfusion changes in the right lingual gyrus, previously linked to VSS pathophysiology,^9 48^ other parts of the extrastriate visual cortex—the left fusiform gyrus in particular—showed increased activation, confirming the importance of these associative areas in the syndrome.^49^ This mismatch could be attributed to differences in spatial resolution between [¹⁸F]FDG PET and MRI. Further, while several publications have highlighted a degree of correlation between regional cerebral metabolic rate of glucose metabolism measured by PET and regional CBF, it is not possible to assume that the same correspondence can be detected in subjects with pre-established conditions such as visual snow.

Intuitively, it would have been expected for the visual stimulation to have a bigger impact on the control group, since visual snow patients were more ‘familiar’ with it. Nonetheless, as can be seen from the analysis (online supplemental eFigure 1) conducted to assess the effect of the stimulus, this produced a very similar cortical activation in both groups. Hence, it is possible to speculate that the internal percept of visual snow follows different neuronal pathways respect to that of incoming sensory stimuli.

Finally, the increase in blood flow in the primary auditory cortex may, at least in part, be linked to the high levels of concomitant tinnitus seen in our patients.^50^ The comorbidity between VSS and tinnitus seems to suggest an underlying pathophysiological mechanism common to both conditions, possibly a widespread network phenomena not limited to the involvement of the relevant primary sensory cortices, ultimately causing phantom perceptions.^51^

### Limitations

The main limitation of this study lies in the challenges of investigating VSS while taking into account its main comorbidity of migraine. The high association between the two conditions, confirmed in our patient cohort, hinders the generalisability of results in this population. On the other hand, specifically selecting VSS patients with no concomitant migraine could result in a selection bias, and a patient group not representative of the full condition.

Although this was performed *post-hoc*, the comparative analyses of our two subject cohorts with a group of migraineurs without VSS, and further of the subselection of VSS patients without concomitant migraine compared with controls, showed mostly overlapping results to our original analysis. In the future, these issues will need to be addressed in the preliminary phases of original studies directed at investigating the VSS.

### Conclusions

In conclusion, patients with VSS present increased activation in a wide network of intrinsic brain areas that are key in the processing of complex sensory and cognitive states. The fact that rCBF increases were independent of the presence of an external visual stimulus, suggests that these abnormalities could be a causal factor of the disorder.

This study expands on previous neuroimaging findings, confirms VSS to be a complex brain problem, and helps to improve our understanding of a condition for which treatment is still lacking.

## Data Availability

Data are available upon reasonable request. Data are available from the corresponding author upon reasonable request.
