# Supplementary material for: Localised increase in regional cerebral perfusion in patients with visual snow syndrome: a pseudo-continuous arterial spin labelling study
Source: J Neurol Neurosurg Psychiatry. 2021 Jul 14;92(9):918–26. doi: 10.1136/jnnp-2020-325881 (PMC8372400; doi:10.1136/jnnp-2020-325881)
Supplement: Supplementary data [file jnnp-2020-325881supp001.pdf]

Localised increase in regional cerebral perfusion in patients with visual snow syndrome. A pseudo-continuous arterial spin labelling study.

Supplementary material

1. **Image pre-processing**
2. **Evaluations of visual snow simulation**
3. **Additional pCASL analyses**

   Global CBF analysis

   Evaluation of motion parameters

   F-tests

   Stimulus effects analysis

   eFigure 1: Stimulus effects in VSS patients and Ctrls
4. **References**

Puledda F, *et al. J Neurol Neurosurg Psychiatry* 2021; 92:918–926. doi: 10.1136/jnnp-2020-325881

1. Image pre-processing

Pre-processing of pCASL images was performed using Automated Software for ASL Processing (ASAP (1)) which employs the SPM software suite, version 12 (SPM 12; www.fil.ion.ucl.ac.uk/spm/). Computation of CBF maps was performed by the scanner computer using the following formula:

$$CBF = 600 \frac{e^{w/T_{1a}}}{2\varepsilon T_{1a}(1 - e^{-\tau/T_{1a}})} \frac{P}{\frac{R}{\lambda}}$$

in which P is the signal in the perfusion-weighted image (control–label), R is the signal in the reference image, ε is the combined efficiency of labelling and background suppression (~65%), τ is the label duration (1825 ms), T_{1a} is the T_1 of arterial water, and w is the postlabelling delay (2025 ms). For spatial normalization of the CBF maps to the space of the MNI within the ASAP framework, a multistep approach was used: CBF maps were co-registered to the T1-weighted structural ADNI images, after coarse alignment of the origin of both images. Unified segmentation of the T1-weighted image normalised this image to the MNI space and was used to produce a 'brain-only' binary mask which was multiplied by the co-registered rCBF map to produce an image free of extra-cerebral artefacts. The spatial transformation matrix was applied to the clean CBF images and then smoothed using an 8x8x8 mm Gaussian kernel. To measure global CBF signal, the ASAP toolbox was used to extract average CBF values from a grey matter mask for each subject. Probabilistic grey matter images in MNI space, derived from the FSL voxel-based morphometry toolbox, were thresholded to produce a mask which included all voxels with a >20% likelihood of being grey matter. A global CBF value, defined as the mean of all grey matter voxels within the mask, was computed for each individual pCASL CBF map. Mean global CBF for VSS patients and Ctrls in both conditions were compared with standard t-test. Global CBF differences were tested in an ANCOVA model, while controlling for the underlying experimental condition (i.e. baseline vs snow-like stimulus).

Puledda F, *et al.* *J Neurol Neurosurg Psychiatry* 2021; 92:918–926. doi: 10.1136/jnnp-2020-325881

2. Evaluations of visual snow simulation

A comparison between the ‘snow-like’ visual simulation used as a task for the MRI sequence and the personal experience of visual snow itself, was collected from each VSS patient at the end of the scanning session. The patients were asked to rate the similarity of the simulation to their own visual static percept by rating the following parameters: density, speed, size and colour. The visual static percept could be classified as equal (=) increased (>) or reduced (<) respect to the simulation. Patients could also describe their visual static as being more black and white (BW), transparent (T), flashing (F) or coloured (C), respect to the simulation. The evaluation for each patient is shown in the table below.

Patient ID	Static Density	Static Speed	Size	Colour
1	<	<	<	C
2	=	>	=	=
3	=	=	=	=
4	=	=	<	F
5	=	=	=	=
6	=	=	=	=
7	=	=	=	=
8	=	=	=	=
9	=	=	=	T
10	>	=	<	=
11	>	=	=	T
12	>	=	<	=
13	=	=	<	T
14	=	=	<	=
15	=	<	=	=
16	=	=	=	T
17	=	=	=	=
18	=	=	<	C
19	=	=	<	=
20	=	=	=	=
21	>	=	=	=
22	>	=	<	T
23	=	=	=	=
24	=	=	=	T

3. Additional pCASL analyses

Global CBF analysis

Mean global CBF in grey matter values did not differ between groups, whether measured at baseline (mean ± standard error 53.5 ± 10.6 in the VSS group and 51.7 ± 12 in Ctrls; *P* = 0.6) when looking at the snow-like stimulus (54.9 ± 10.5 in VSS and 53.3 ± 12.8 in Ctrls; *P* = 0.6) or averaging both conditions (54.2 ± 10.5 in the VSS group and 52.5 ± 12.3 in Ctrls; *P* = 0.5). An ANCOVA analysis confirmed no significant differences in median global CBF values between groups, when accounting for stimulus type.

Evaluation of head motion

Cumulative distance travelled was analysed as a measure of head motion, and showed no significant differences between the two groups (0.17 ± 0.12 in the VSS group and 0.16 ± 0.09 in Ctrls; *P* = 0.8).

F-tests

An F contrast to evaluate the main effect of group on whole-brain CBF maps showed a significant increase in regional CBF in the same nine clusters presented in the main analysis (Table 1), with the following F and k values:

**Brain region**	*F*	k	x	y	z
Cuneus and precuneus	28.81	362	52	-54	54
Cuneus and precuneus	22.08	1000	-26	-82	32
Superior temporal gyrus	21.35	147	-54	-30	14
Precentral gyrus	21.25	460	-46	0	46
Precentral gyrus	18.89	279	44	0	48

Cerebellum and fusiform gyrus	17.78	139	-38	-56	-22
Inferior parietal lobule	16.66	462	-64	-40	42
Posterior cingulate gyrus	16.51	129	-4	-40	46
Supplementary motor area	14.57	210	2	10	52

Stimulus effects

When subject to the snow-like stimulus and compared to baseline (i.e. resting fixation), both groups showed an increase in rCBF in a large cluster involving the primary visual cortex, lingual gyrus and inferior temporal gyrus. In VSS patients only, the stimulation also evoked a decrease in rCBF in the mid-cingulate and posterior cingulate cortex. eFigure 1 shows these areas of increased and decreased rCBF in patients and controls, when subject to the snow-like stimulus.

eFigure 1: Stimulus effects in VSS patients and Ctrls

Areas of increases (red/yellow) and decreases (blue/green) of rCBF in VSS patients (a) and Ctrls (b) when subject to the snow-like stimulus. All areas are significant at the cluster level whole-brain analyses and corrected for cluster extent. Bars represent *T*-values. For deactivation cluster in a) k = 551; MNI coordinates: x = 12 y = -10 z = 46.

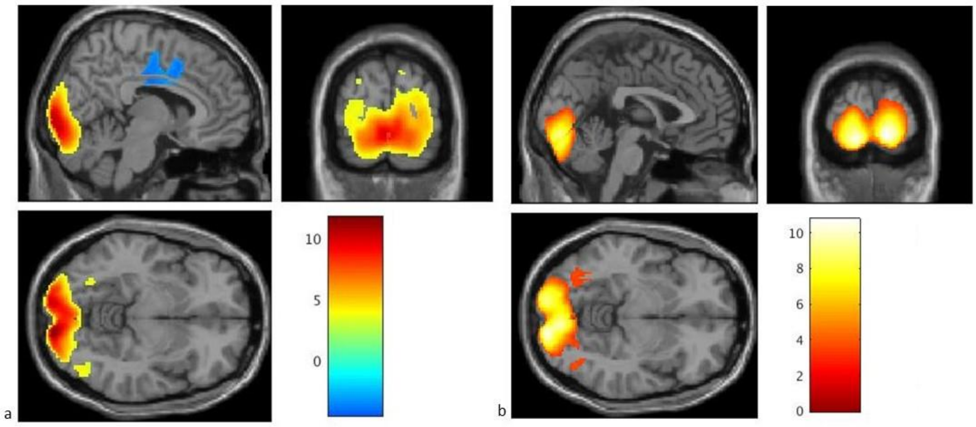

Puledda F, *et al. J Neurol Neurosurg Psychiatry* 2021; 92:918–926. doi: 10.1136/jnnp-2020-325881

4. References

1. Mato Abad V, Garcia-Polo P, O'Daly O, Hernandez-Tamames JA, Zelaya F. ASAP (Automatic Software for ASL Processing): A toolbox for processing Arterial Spin Labeling images. Magnetic resonance imaging. 2016;34(3):334-44.
